# Bimodal microwave and ultrasound phantoms for non-invasive clinical imaging

**DOI:** 10.1038/s41598-020-77368-5

**Published:** 2020-11-23

**Authors:** Enrique Villa, Natalia Arteaga-Marrero, Javier González-Fernández, Juan Ruiz-Alzola

**Affiliations:** 1 IACTEC Medical Technology Group, Instituto de Astrofísica de Canarias, San Cristóbal de La Laguna, 38205 Spain; 2 Department of Biomedical Engineering, Instituto Tecnológico de Canarias, Santa Cruz de Tenerife, 38009 Spain; 3 Department of Signals and Communications, University Research Institute for Biomedical and Health Research, Universidad de Las Palmas de Gran Canaria, Las Palmas de Gran Canaria, 35016 Spain

**Keywords:** Medical research, Electrical and electronic engineering, Biotechnology, Tissues

## Abstract

A precise and thorough methodology is presented for the design and fabrication of bimodal phantoms to be used in medical microwave and ultrasound applications. Dielectric and acoustic properties of human soft tissues were simultaneously mimicked. The phantoms were fabricated using polyvinyl alcohol cryogel (PVA-C) as gelling agent at a 10% concentration. Sucrose was employed to control the dielectric properties in the microwave spectrum, whereas cellulose was used as acoustic scatterer for ultrasound. For the dielectric properties at microwaves, a mathematical model was extracted to calculate the complex permittivity of the desired mimicked tissues in the frequency range from 500 MHz to 20 GHz. This model, dependent on frequency and sucrose concentration, was in good agreement with the reference Cole–Cole model. Regarding the acoustic properties, the speed of sound and attenuation coefficient were employed for validation. In both cases, the experimental data were consistent with the corresponding theoretical values for soft tissues. The characterization of these PVA-C phantoms demonstrated a significant performance for simultaneous microwave and ultrasound operation. In conclusion, PVA-C has been validated as gelling agent for the fabrication of complex multimodal phantoms that mimic soft tissues providing a unique tool to be used in a range of clinical applications.

## Introduction

Phantoms are considered artificially produced materials that mimic human tissues providing well-known geometry and material composition^1^. They have been widely used as test models for a variety of peripheral tissue imaging techniques and image-guided interventions as well as medical training purposes^2,3^. Furthermore, phantoms designed to evaluate multimodal techniques, such as photoacoustic imaging, elastography-based imaging, and percutaneous needle insertion^2,4^, are of particular interest. This is because the combination of several imaging techniques provides advanced diagnostic results^5^. However, specific requirements are on demand, and although commercially-available phantoms are usually designed for specific applications or broad markets, customized alternatives are not often provided^6^.

Polyvinyl alcohol cryogel (PVA-C) provides textural and elastic properties quite similar to biological tissues and it is easily customized at low-cost. The resulting PVA-C-based phantoms have high structural rigidity, indefinite longevity^7,8^, and are more resistant to crack formation in comparison to agar- and gelatin-based phantoms^6^. The biggest advantages are their biocompatibility and hydrophilicity which provide protein absorption capabilities and support cellular growth^9,10^. Oppositely, their most critical disadvantage is the fabrication process, since a thorough and precise temperature control is required as well as a long post-manufacturing time depending on the number of freeze-thaw cycles^6,9^. PVA-C tissue phantoms have been employed before for ultrasound (US) elastography providing a non-invasive and non-destructive method to investigate the elastic properties of healthy and pathological soft tissues^9,11,12^.

We are focused on the establishment of a clinical workflow in which external and internal temperature measurements are combined with diagnostic ultrasound. Initially, an infrared sensor^13^ provides the external temperature by acquiring surface images and anomalous temperature patterns can be detected upon inspection and analysis. These specific areas of interest with anomalies are further analyzed by subcutaneous, in-depth temperature measurements using microwave technology guided by US imaging. Therefore, tissues under evaluation are properly assessed regarding in-depth temperatures and acoustic features while significant information is gathered to achieve medical diagnosis. The development of multimodal phantoms is required to assess and validate the complementary microwave and US measurements. The described workflow could be very useful in cutaneous lesions, such as diabetic foot ulcers, sarcoma, melanoma or even to delineate the extension of venous thrombosis, as well as to guide re-perfusion and re-vascularization procedures^14^. Tumoral or inflammatory lesions in organs like lung, liver or intestines, would be aided by such workflow in which an anomalous temperature pattern may be a warning sign^15–17^. In particular, our aim is to develop a clinical protocol to detect diabetic neuropathies for a personalised diagnosis and treatment monitoring. In this regard, infrared sensors have previously demonstrated their viability to analyze anomalous temperature patterns in diabetic diseases^18–22^.

The development of PVA-C phantoms has been successfully demonstrated for wideband operation at microwave frequencies in the range from 500 MHz to 20 GHz^23^. These phantoms were developed to characterize a microwave device for a medical application focused on the detection and monitorization of diabetic neuropathy. Since clinically, vascular sufficiency in this pathology is assessed using US Doppler effect^24^, the aim of the present work was to extend the phantoms’ use to an additional medical imaging modality being US. Thus, the temperature measured by the intended microwave-device would be simultaneously complemented with the US images, providing multimodal capabilities to the phantoms. The inclusion of complex internal structures into these bimodal phantoms, such as blood vessels or capillaries, may improve the described workflow providing a more specific diagnosis.

In this work, a full methodology is proposed for the fabrication of custom phantoms with specific properties for a bimodal medical application based on microwave and US imaging. A set of phantoms at 10% PVA-C and 1% cellulose concentrations was manufactured employing a varying sucrose concentration (0%, 10%, 20%, 30%, 40%, 50% and 60%). A mathematical model dependent on frequency (from 500 MHz to 20 GHz) and sucrose concentration was fitted to enable the adjustment of the complex permittivity of the desired mimicked tissues in the microwave band. The fitted model was validated by comparison to the theoretical Cole–Cole model^25–28^. In addition, US capabilities were assessed and analyzed to validate the additional modality in terms of scatterers’ concentration, image quality as well as acoustic properties such as the speed of sound and attenuation coefficient.

## Material and methods

### Phantom preparation

PVA-C is a weak acoustic attenuator^2,29^. Thus, the addition of a material or particles which produce a strong attenuation was required. These added materials may affect, to some extent, the mechanical properties of PVA-C. Several alternatives were found in the literature to control the acoustic properties which include the speed of sound, the attenuation coefficient and the backscatter coefficient. Among other materials, n-propanol and propylene glycol were used to modify the speed of sound; while evaporated milk, cellulose, aluminum oxide ($\mathrm{{Al}}_{2}\mathrm{{O}}_{3}$), talcum powder, silicon carbide (SiC) and graphite were employed as scattering agents to vary the attenuation^6,29,30^. Dimethyl sulfoxide (DMSO) was usually added to facilitate structural arrangement in the solution, lowering the freezing point and strengthening the gel^31^. Furthermore, glass beads and cellulose were also employed as scatterers in PVA-C phantoms^7,9^ and the main alternatives considered in the present work. However, cellulose was found more suitable during the fabrication process, since it was easily mixed and provided the most replicable results.

A set of seven phantoms were fabricated using PVA-C (99% hydrolyzed, molecular weight 89000-98000, Sigma Aldrich, 10% concentration) as gelling agent, cellulose as US scatterer (Cellulose microcrystalline, 20 $\mu$m, Sigma Aldrich, 1% concentration) and a variable concentration of sucrose, from 0% up to 60% in steps of 10%, in the form of table sugar (99% purity). The corresponding percentages required for phantom fabrication were calculated in weight/weight (w/w) considering a final mix of 120 g per phantom. In a container continuously heated and magnetically stirred, distilled water, sucrose and benzoic acid (Sigma Aldrich, 0.1%, equivalent to 0.12 g), employed for preservation purposes, were mixed until the solutes have completely dissolved prior to the addition of PVA-C (12 g). The container was covered to minimize dehydration. Subsequently, heat was retired when the mixture acquired a homogeneous consistency, and cellulose was added. The solution was mixed purposely for a couple of minutes and its weight was corrected by adding distilled water which may have evaporated during the heating process. Finally, the mixture was poured in labelled plastic containers in a slow and controlled manner, minimizing the amount of air bubbles. No vacuum pump was used to eliminate remaining air bubbles, instead samples were allowed to naturally cool down at a room temperature of 25 $^\circ$C, for approximately 30 minutes, to establish a uniform temperature before freezing. It must be noted that identical containers were used for each fabricated phantom, that is, each had the same composition and geometry, as well as the same volume of mixture. The 30 minutes period is an indicative value, since the actual aim is to achieve a uniform ambient temperature. Thus, the same temperature slope was expected for all the phantoms when introduced in the freezer ($-20\,^\circ$C) during 12 h to complete the freeze-thaw cycle required for the cross-linking of the polymer. Finally, the phantoms were thawed in the fridge at 2 $^\circ$C for 24 h and stored there until measurement.

The influence of the number of freeze-thaw cycles on the speed of sound is controversial. Some studies reported an increasing logarithmic relationship between the number of cycles and the speed of sound^9^, whereas others noticed a minor effect of the number of cycles for both, the speed of sound and attenuation coefficient^29^. Additionally, a high number of cycles was associated with a tendency to dehydrate^9^. Therefore, a single freeze-thaw cycle was selected.

The percentage of the scatterer material has reportedly little impact on the speed of sound^9^. Nevertheless, a set of phantoms were fabricated for testing purposes using a varying concentration of cellulose. As can be observed in the Experimental results section (Table 3), the speed of sound was approximately constant between 0% and 2% concentration for 18-mm thickness phantoms. Therefore, for simplicity, cellulose was added at 1% concentration.

The long-term stability and longevity are sustained by the benzoic acid instead of involving a saline solution that may modify the dielectric properties of the phantoms, particularly their conductivity. In fact, the stability and extended shelf-life of these phantoms were previously demonstrated^23^ and their properties were not significantly changed after a 7-month time span.

### Data acquisition

#### Microwave data and model extraction

**Figure 1:**
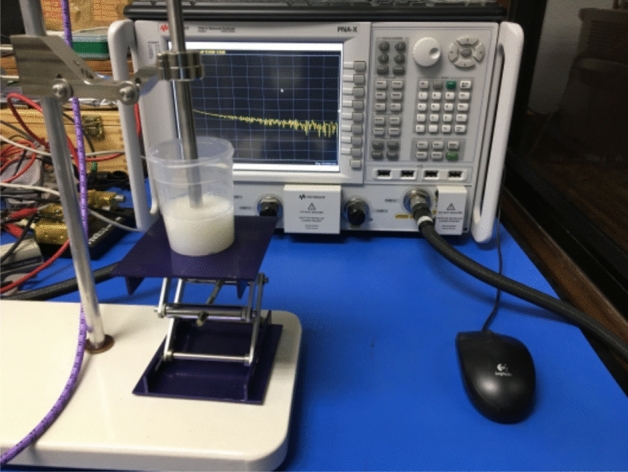
Experimental setup for the characterization of the dielectric properties of each fabricated phantom.

The dielectric properties of the phantoms were characterized using a well-known measurement protocol based on the use of an open-ended coaxial probe^32,33^ Performance Probe model 85070E and a vector network analyzer model N5245A, both from Keysight Technologies. This setup is shown in Fig. 1. The measurement protocol was thoroughly reported previously, as well as the extracted mathematical model to characterize 15% PVA-C phantoms based on frequency and sucrose concentration^23^. This model provides a design methodology to match the desired real and imaginary parts of the dielectric relative permittivity, $\varepsilon _{r}^{'}$ and $\varepsilon _{r}^{''}$. Notice that, in comparison with the previous study, the modification of the PVA-C concentration employed in the current fabrication (10%) and the addition of the acoustic scatterers (1%) require a new parametrization.

Shortly, experimental data ($\varepsilon _{r}^{'}$ and $\varepsilon _{r}^{''}$) were fitted to a third-order polynomial as a function of frequency, *f* (GHz), for each phantom with a fixed sucrose concentration, ${S_c}$, in the frequency band from 500 MHz to 20 GHz. Considering the real part of the permittivity, the expression is as follows:

1 $$\begin{aligned} \varepsilon _{r}^{'} = a_{\varepsilon ^{'}}\cdot f^{3} + b_{\varepsilon ^{'}}\cdot f^{2} + c_{\varepsilon ^{'}}\cdot f + d_{\varepsilon ^{'}} \end{aligned}$$

where *a*, *b*, *c*, and *d* are a set of fitting parameters dependent on sucrose concentration, ${S_c}$. These parameters are subsequently fitted to a quadratic function, following a similar approach as previous studies^27,34^. Thus, Eq. (1) was transformed in terms of ${S_c}$ as follows:2An analogous procedure applied to the complex part of the permittivity, $\varepsilon _{r}^{''}$, allowed the extraction of the corresponding parameters (from $A_{\varepsilon ^{''}}^{'}$ to $D_{\varepsilon ^{''}}^{'''}$). The dielectric conductivity can be derived from the imaginary part of the permittivity using the following expression^27,35^:

3 $$\begin{aligned} \sigma = 2 \cdot \pi \cdot f \cdot \varepsilon _{o} \cdot \varepsilon _{r}^{''} \end{aligned}$$

Lastly, the experimental data were fitted to the Cole–Cole model^25^, a commonly-used reference to describe the dielectric constant as a function of frequency. The procedure employed has been previously described^23^.

#### Ultrasound data acquisition

The speed of sound and the acoustic attenuation are considered significant phantom properties (typically in the frequency range from 3 to 22 MHz)^1,36^ which were quantitatively and precisely characterized in the present study.

**Figure 2:**
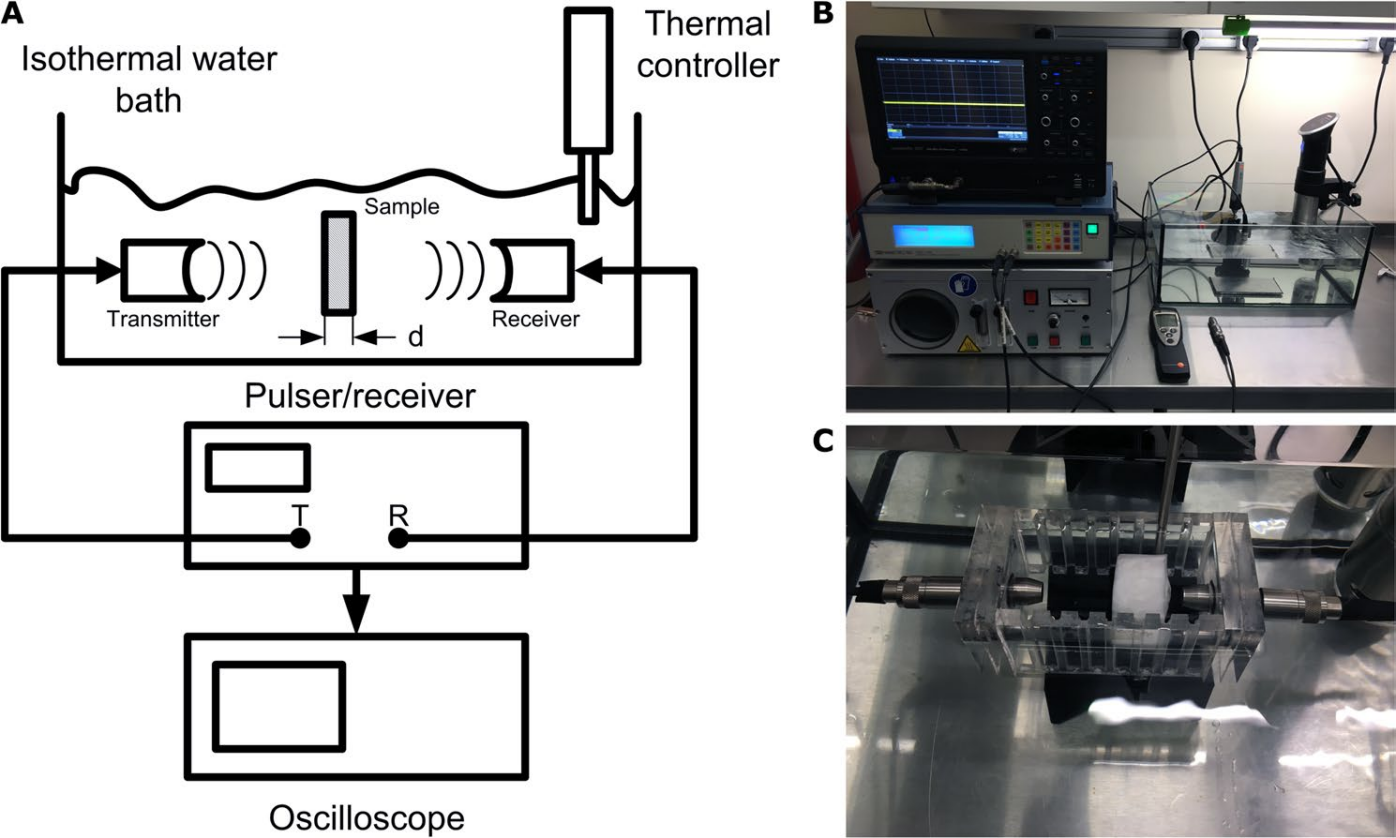
Experimental setup employed for the ultrasound experiments: (**A**) Scheme of through-transmission configuration for ultrasonic spectroscopy. (**B**) General view of equipment. (**C**) Detailed view of the phantom under measurement.

The experimental setup consisted of a two-stage process. First, US images were acquired using a commercial device (MicrUs EXT-1H, Telemed UAB) to assess qualitatively the images. The acoustic properties of the fabricated phantoms were then measured by a well established method, the through-transmission ultrasonic spectroscopy^3,37–39^. Shortly, a computer controlled pulser/receiver (Panametrics-NDT 5800) was combined with two broadband transducers, which were positioned, as indicated in Fig. 2A, inside a water bath stabilized at approximately 25 $^\circ$C. A fixed frequency of 5 MHz was employed for the emitting transducer. Two ultrasonic pulse signals were separately recorded in an oscilloscope, one without any material in between the transducers, $S{_w}$ taken as reference signal, and another with the material to characterize in between, $S{_p}$. The measurements were taken in a three-day period and each recorded signal was averaged over one thousand acquisitions. Thus, the acoustic velocity and the attenuation were obtained for each fabricated phantom taking water as reference.

The experimental setup used is shown in Fig. 2, in which the employed equipment and configuration for a phantom measurement are displayed. Regarding the speed of sound, this method requires to determine the thickness of the phantom, *d*, and the speed of sound in water, $V_{0}$, which is a function of the temperature, *T* ($^\circ$C), as expressed in the following equation^36,40^:

4 $$\begin{aligned} V_{0} = 1403 + 5\cdot T - 0.06 \cdot T^{2} + 3 \cdot 10^{-4} \cdot T^{3} \end{aligned}$$

Subsequently, the speed of sound in the sample is calculated as^36,40^

5 $$\begin{aligned} V_{s} = \frac{V_{0}}{1-dt\cdot \left( \frac{V_{0}}{d}\right) } \end{aligned}$$

where *dt* is the difference in transit time between the recorded pulses, consisting of averaged signals over one thousand pulses with each phantom. The thickness of each phantom, *d*, was determined using a standard digital caliper.

The same setup was employed to measure the attenuation coefficient, $\alpha$. In this case, the amplitudes of the signals and the thickness of the phantoms were considered. $\alpha$ was derived from the following equation^41^:

6 $$\begin{aligned} \alpha = -\frac{8.686}{d} \cdot \Big [ ln{\frac{A}{A_{w}}} + ln{\frac{1}{T}} \Big ] \end{aligned}$$

where *A* and $A_{w}$ are the amplitudes of the signals through phantom and water, respectively, and 8.686 is the conversion factor from Neper/cm to dB/cm. *T* is the transmission coefficient for a double boundary problem that can be derived from the following equation^41,42^:

7 $$\begin{aligned} T = \frac{4 \cdot z \cdot z_{w}}{(z+z_{w})^2} \end{aligned}$$

in which $z_{w}$ and *z* are the impedances in water and phantom, respectively. These impedances are calculated through the expression^3,41,42^:

8 $$\begin{aligned} z = \rho \cdot V \end{aligned}$$

where $\rho$ represents the density and *V* the speed of sound. Each density was measured by immersing the phantom in two immiscible liquids until a floating equilibrium was reached^43^. A glycerol aqueous solution was employed^44,45^ which allowed us to estimate the density of porous materials ranging between the density of water (1000 kg/m$^{3}$) and that of pure glycerol (1260 kg/m$^{3}$, CAS number 56-81-5) at room temperature (25 $^\circ$C). The density of the phantom containing 50% sucrose was calculated by weight and volume, since its density exceeded the limits of the floating method. Thus, the standard deviation was higher for this phantom. The resulting densities are listed in Table 4, in the Experimental results section.

Subsequently, frequency-dependent signal loss was determined for the set of fabricated phantoms, except the 60% sucrose concentration phantom due to limited consistency. For each phantom corresponding to each sucrose concentration (from 0 to 50%), the fast Fourier transform (FFT) of each time-domain averaged trace was calculated to obtain the one-sided power spectral density^39^ estimated up to 10 MHz.

Operator’s dependence was not observed in the employed measurement method to derive the acoustic properties. The operator’s duty was limited to introduce and extract each phantom in the measurement chamber as well as to start and finish the data acquisition. Furthermore, the data analysis was performed using MATLAB^46^ scripts to derive the US parameters from the respective measurements.

## Experimental results

### Microwave data and model extraction

**Figure 3:**
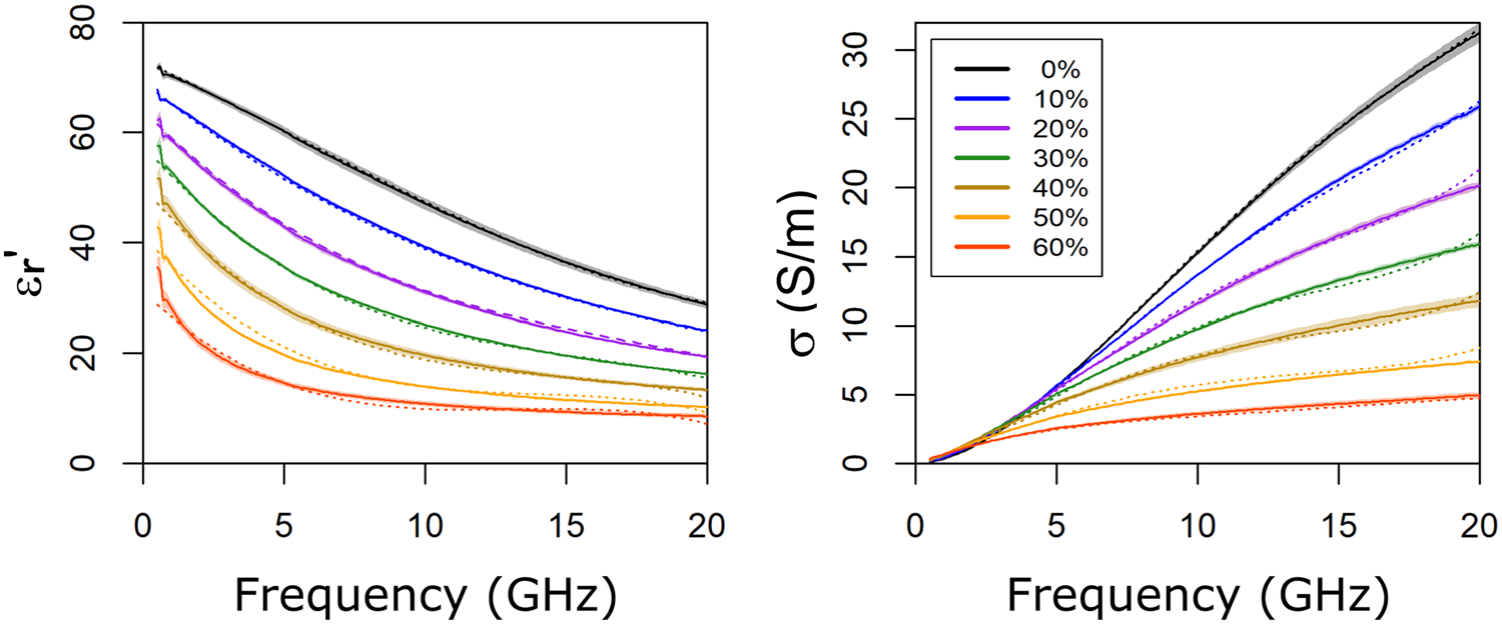
Average values of the real part of the relative permittivity ($\varepsilon _{r}^{'}$) and conductivity ($\sigma$) versus frequency, as well as the mathematical model extracted, according to the varying sucrose concentration. The curves are color-coded depending on the sucrose concentration as seen in the legend. The solid lines represent the mean value whereas the dotted lines represent the extracted model. The shaded areas correspond to the standard deviation associated with the experimental data.

Figure 3 displays the relative permittivity and conductivity of each phantom as a function of the sucrose concentration employed in the fabrication. In this figure, all the measurements taken in a ten-day period are summarized. The mathematical model extracted is also displayed.

The accuracy provided by this measurement protocol was within 4% and 7% for the real and imaginary parts of the complex permittivity ($\varepsilon _{r}^{'}$ and $\varepsilon _{r}^{''}$), respectively, whereas repeatabilities were both within 0.5% in the considered frequency range^23^. Regarding the performance of the bimodal phantoms in the microwave range, the results indicate that the model extracted fits properly the experimental data. A direct comparison between the extracted model in a previous work^23^ and the present one can only be performed at certain sucrose concentrations, that is, 0%, 30% and 60%.

The goodness-of-fit was based on the pseudo coefficient of determination ($R^{2}$) defined as

9 $$\begin{aligned} R^{2} = 1 - \frac{RSS}{TSS} = 1 - \frac{\sum (y_{i}-f_{i})^{2}}{\sum (y_{i}-\overline{y})^{2}} \end{aligned}$$

where $y_{i}$ corresponds to the experimental data, $\overline{y}$ its mean value at each frequency point and $f_{i}$ the fitted model values. *RSS* is the residual sum of squares calculated as the deviation predicted from actual empirical values of data and *TSS* is the total sum of squares between the observations and their overall mean. The lowest $R^{2}$ for the real part of the permittivity was found for the 60% concentration, being 0.970, whereas for the imaginary part, it was 0.916 for a 40% sucrose concentration. Overall, the variations in $R^{2}$, according to sucrose concentration, were around 1% and 4% for the real and the imaginary parts of the permittivity, respectively. The homogeneity of the measurements, in terms of the percentage of variation, was below 4% for both parts of the permittivity in the considered frequency range. In comparison with the previously extracted model at 15% PVA-C concentration (without US scatterers)^23^, the fitting errors were quite similar with variations around 1% and 3% for the real and the imaginary parts of the permittivity, respectively. In this case, the homogeneity of the measurements was below 2%.

The corresponding parameters defining the model extracted, which allow us to obtain the real and the imaginary part of the relative permittivity, are presented in Table 1. For the real part of the permittivity, $R^{2}$ presented the lowest value for the parameters *C*; whereas for the imaginary part, $R^{2}$ was the lowest for the *A* parameters, being 0.991 and 0.992, respectively.

**Table 1:** Parameters to obtain the real ($\varepsilon ^{'}_{r}$) and the imaginary ($\varepsilon ^{''}_{r}$) parts of the relative permittivity according to the sucrose concentration used.

Parameters	$\varepsilon ^{'}_{r}$	Parameters	$\varepsilon ^{''}_{r}$
$A_{\varepsilon ^{'}}^{'}$	3.978$\times 10^{-6}$	$A_{\varepsilon ^{''}}^{'}$	$-4.963\times 10^{-6}$
$A_{\varepsilon ^{'}}^{''}$	$-4.466\times 10^{-4}$	$A_{\varepsilon ^{''}}^{''}$	1.920$\times 10^{-4}$
$A_{\varepsilon ^{'}}^{'''}$	2.673$\times 10^{-3}$	$A_{\varepsilon ^{''}}^{'''}$	5.184$\times 10^{-3}$
$B_{\varepsilon ^{'}}^{'}$	$-1.810\times 10^{-4}$	$B_{\varepsilon ^{''}}^{'}$	1.582$\times 10^{-4}$
$B_{\varepsilon ^{'}}^{''}$	1.803$\times 10^{-2}$	$B_{\varepsilon ^{''}}^{''}$	$-3.768\times 10^{-3}$
$B_{\varepsilon ^{'}}^{'''}$	$-4.343\times 10^{-2}$	$B_{\varepsilon ^{''}}^{'''}$	$-0.279$
$C_{\varepsilon ^{'}}^{'}$	2.496$\times 10^{-3}$	$C_{\varepsilon ^{''}}^{'}$	$-1.028\times 10^{-3}$
$$C_{\varepsilon ^{'}}^{''}$$	$$-0.193 $$	$$C_{\varepsilon ^{''}}^{''}$$	$$-4.037\times 10^{-2}$$
$$C_{\varepsilon ^{'}}^{'''}$$	$$-2.393 $$	$$C_{\varepsilon ^{''}}^{'''}$$	4.796
$D_{\varepsilon ^{'}}^{'}$	$-6.329\times 10^{-3}$	$D_{\varepsilon ^{''}}^{'}$	$-1.589\times 10^{-3}$
$$D_{\varepsilon ^{'}}^{''}$$	$$-0.316 $$	$$D_{\varepsilon ^{''}}^{''}$$	0.290
$D_{\varepsilon ^{'}}^{'''}$	72.972	$D_{\varepsilon ^{''}}^{'''}$	2.474

Regarding the Cole–Cole model, Table 2 provides the respective fitting parameters according to the selected sucrose concentration. The lowest $R^{2}$ was found for the $\sigma _{s}$ parameter, being 0.971, while the rest of the parameters presented values above 0.993. In comparison with the previously extracted model, the worse fitted parameter then was $\Delta \varepsilon$. In the present model, $\sigma _{s}$ had to be fitted to a higher degree polynomial (fourth order), and even though, the fitted values were lower than previously reported.

**Table 2:** Cole–Cole parameters dependent on the sucrose concentration for the fabricated phantoms.

Parameters	PVA-C
$\varepsilon _\infty$	$-5.049\times 10^{-5}\times S_{c}^3$ + 4.682$\times 10^{-3}\times S_{c}^2 \,- 5.238 \times 10^{-2}\times S_{c}$ + 3.159
$\Delta \varepsilon$	$-1.933\times 10^{-3}\times S_{c}^2\, -3.905\times 10^{-1}\times S_{c}$ + 68.300
$\tau$	1.229$\times 10^{-5}\times S_{c}^4\, - 5.751 \times 10^{-4}\times S_{c}^3$ + 1.744$\times 10^{-2}\times S_{c}^2$ + 1.493$\times 10^{-1}\times S_{c}$ + 10.800
$\sigma _{s}$	$-4.981\times 10^{-8}\times S_{c}^4$ + 6.280$\times 10^{-6}\times S_{c}^3$ $- 2.299\times 10^{-4}\times S_{c}^2$ + 1.670$\times 10^{-3}\times S_{c}$ + 5.675$\times 10^{-2}$
$\alpha$	5.417$\times 10^{-8}\times S_{c}^4 \, - 5.956\times 10^{-6}\times S_{c}^3$ + 1.327$\times 10^{-4}\times S_{c}^2$ + 4.884$\times 10^{-3}\times S_{c}$ + 1.026$\times 10^{-1}$

### Ultrasound data

**Figure 4:**
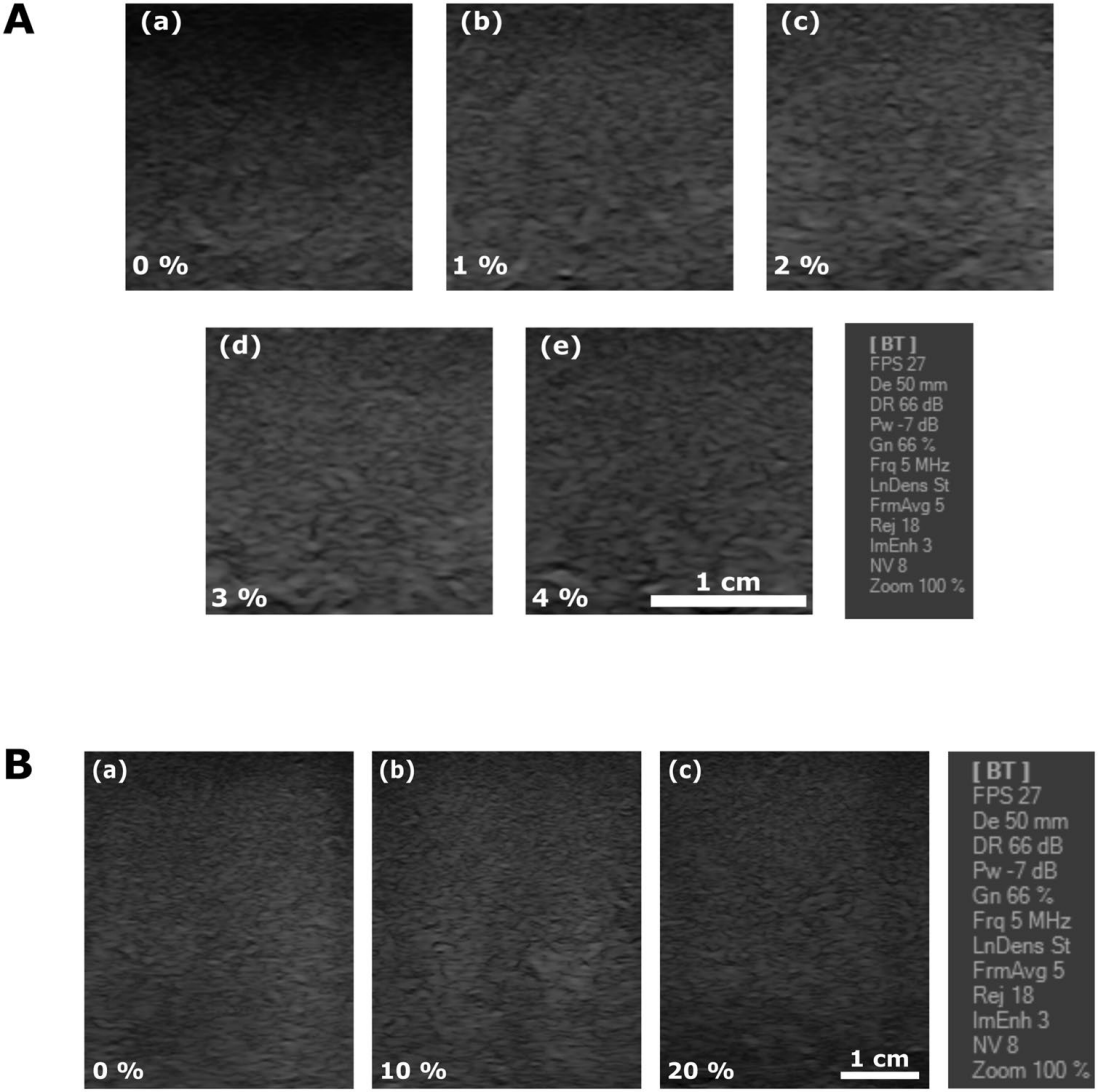
US images acquired at varying cellulose concentration without added sucrose (Panel **A**): (**a**) 0%, (**b**) 1%, (**c**) 2%, (**d**) 3%, and (**e**) 4%; as well as at varying sucrose concentration for a fixed 1% cellulose (Panel **B**): (**a**) 0%, (**b**) 10%, and (**c**) 20%.

Once the microwave measurements were completed, qualitative and quantitative US characterization was performed. For illustrative purposes, images acquired for various cellulose and sucrose concentrations are displayed in Fig. 4, panel A and B, respectively. A linear probe model L12-5L40S-3 Telemed UAB was employed, using the following settings: 5 MHz frequency, 50 mm depth, focus between 10 and 14 mm, $-7$ dBm power, 66 dB dynamic range and 66% gain, as indicated in Fig. 4.

The concentration of PVA-C employed in the fabrication has been lowered, in comparison to our previous work^23^, to better match the speed of sound. In fact, a decrement in the speed of sound was confirmed, being 1540.4 m/s and 1576.9 m/s, for 10% and 15% PVA-C concentration, respectively.

Table 3 lists the dependence of the speed of sound on cellulose concentration. The addition of acoustic scatterers at varying concentration did not modify significantly the speed of sound, which was practically constant from 0% to 2%, and increased slightly for concentrations between 3% and 4%, as listed.

**Table 3:** Measured speed of sound versus concentration of cellulose employed in the phantom fabrication.

Cellulose concentration (%)	Speed of sound (m/s)
0	1540.4 ± 0.2
1	1540.3 ± 0.3
2	1540.2 ± 0.1
3	1547.3 ± 0.3
4	1547.7 ± 0.1

**Figure 5:**
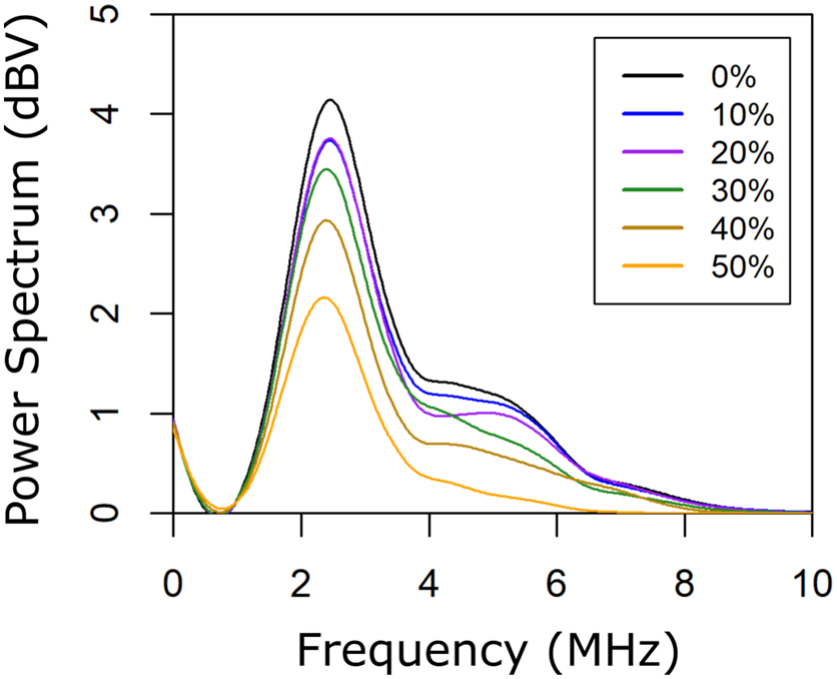
Power spectrum versus frequency for varying sucrose concentration of the fabricated phantoms.

A summary of the measured density (kg/m$^{3}$), speed of sound (m/s) and attenuation (${\text{dB}/\text{cm} \cdot \text{MHz}}$), in addition to their corresponding standard deviations, are listed in Table 4 in which variations associated with the increment in sucrose concentration can be observed. As in most human tissues, acoustic attenuation increases with frequency following a power law function^2^. For the US measurements, repeatability and homogeneity were below 0.3% and 0.2%, respectively, for the speed of sound whereas the corresponding values for the attenuation coefficient were below 9% and 7%. Figure 5 displays the power spectrum according to the sucrose concentration in order to observe its effect. It can also be noted the increased attenuation caused by increases in sucrose.

**Table 4:** Density, speed of sound and attenuation dependence on the sucrose concentration for the fabricated phantoms (through-transmission configuration).

S$_{c}$ (%)	Density (kg/m$^{3}$)	Speed of sound (m/s)	Attenuation $({\text{dB}/\text{cm} \cdot \text{MHz}})$
0	1052 ± 3	1536.1 ± 0.1	0.030 ± 0.003
10	1093 ± 3	1577.3 ± 0.1	0.054 ± 0.001
20	1143 ± 2	1616.3 ± 1.8	0.061 ± 0.002
30	1194 ± 3	1685.3 ± 1.0	0.109 ± 0.005
40	1238 ± 5	1749.9 ± 2.7	0.201 ± 0.016
50	1328 ± 11	1819.2 ± 3.9	0.528 ± 0.025

## Discussion

The fabricated phantoms provide improved features, in comparison to our previous study^23^, by accommodating the bimodal functionality. The mathematical model to describe the dielectric properties has been extracted accordingly, although a new parametrization was required due to the lower gelling agent concentration employed in the fabrication. The comparison between the former (15% PVA-C) and the current (10% PVA-C) model indicate a similar performance for the real and the imaginary parts of the permittivity based on the pseudo coefficients of determination ($R^{2}$). With regards to the Cole–Cole model, a higher degree polynomial was required to fit the parameter $\sigma _{s}$ in the current model. In addition, both models, the extracted and the Cole–Cole (data not shown), fit nicely the experimental data. Overall, the capabilities of these phantoms in the microwave range exhibit the same potential as the previously reported ones, without any relevant effect noticed after lowering the gelling agent concentration. However, their capabilities are increased by the addition of the US modality, using scatterers for improved echogenicity.

The performance of the bimodal phantoms in the US modality was also tested. In soft tissues, the average speed of sound is 1540 m/s, which is the value usually assumed in clinical ultrasound scanners^36^, while attenuation coefficients range from 0.4 to 3.3 ${\text{dB}/\text{cm} \cdot \text{MHz}}$^1,47^, being as low as 0.18 ${\text{dB}/\text{cm} \cdot \text{MHz}}$ for blood^1,6^. It must be noted that a differing speed of sound induces image blurring due to non-adjusted beam forming^3^. The phantom fabricated using a 10% PVA-C concentration, without the addition of acoustic scatterers, provides speed of sound values close to those of soft tissues. Therefore, the basis to diminish the PVA-C concentration is supported.

In comparison with previous studies, the speed of sound and attenuation coefficient reported for PVA-C depends on the number of freeze-thaw cycles, ranging between 1520 m/s and 1610 m/s and 0.07 to 0.35 ${\text{dB}/\text{cm} \cdot \text{MHz}}$, respectively^6,36^. Furthermore, 10% PVA-C solution, in one cycle, attained a speed of sound ranging between 1520 and 1560 m/s; and attenuation coefficient between 0.07 and 0.28 ${\text{dB}/\text{cm} \cdot \text{MHz}}$^31^. Our results are within the reported range for the speed of sound and a single cycle provided a good match with soft tissues, this is, 1540 m/s for 10% PVA-C. The concentration of cellulose employed in the fabrication had practically no effect in the speed of sound as previously reported^9^. Thus, the lowest concentration at 1%, used for simplicity, provided a realistic contrast in the ultrasound images acquired. In this manner, the speed of sound remained constant while the attenuation coefficient increased. Regarding the attenuation, the measured values, without the addition of scatterers, were 0.023 and 0.038 ${\text{dB}/\text{cm} \cdot \text{MHz}}$ at 10% and 15% PVA-C concentration, respectively. Thus, the attenuation increased as a function of the PVA-C concentration with measured values in the same order of the previously reported range. The low attenuation coefficient exhibited by the PVA-C for a one freeze-thaw cycle required the addition of acoustic scatterers^2,29^. In fact, at 1% cellulose, although the attenuation coefficient increased, the values were lower than the expected ones. Yet, the addition of sucrose to match the dielectric properties of soft tissues at microwave frequencies produced an increment in the speed of sound as well as in the attenuation coefficient. This effect was also observed with the increment in the number of freeze-thaw cycles^36^. Furthermore, experimental tests confirmed that a 2% cellulose concentration, without added sucrose, approximately doubled the measured attenuation (0.071 ${\text{dB}/\text{cm} \cdot \text{MHz}}$), thus providing values within previously reported ranges for PVA-C.

The discrepancies observed with previously reported values may be due to a series of factors which include the molecular weight of the PVA-C used as well as the fabrication process itself. For sucrose concentrations lower than approximately 20%, the speed of sound was within previously reported ranges. The attenuation coefficients were lower than expected but it can be circumvented by increasing the concentration of scatterers, which, reportedly, do not have a significant impact on the speed of sound. Nevertheless, despite this limitation imposed by the sucrose concentration that can be added to maintain the ultrasound performance within previously reported values, the dielectric properties of soft tissues can still be mimicked. Therefore, the fabricated phantoms fulfilled the requirements for both modalities.

The design and fabrication of custom bimodal phantoms were provided as well as their characterization employing two non-invasive techniques. The flexibility of the customization process and the materials employed even tolerate the inclusion of complex structures within the phantom, such as blood vessels or capillaries, expanding their applicability in the clinical domain. Commercially-available phantoms providing customized alternatives to suit bimodal medical applications are scarce^6^ and the limitations are mainly linked to the applications involved. Commercial solutions that accurately simulate acoustic propagation in tissues can be easily found. However, the heterogeneity within the same soft tissues and inherent differences between patients affect significantly acoustic properties^6^. For these reasons, the design of phantoms that mimic the properties of soft tissues is challenging. The presented phantoms may provide a unique tool to strengthen and promote their use in a range of clinical applications^48^.

## Conclusion

A methodology to design custom bimodal phantoms suitable for clinical microwave and ultrasound applications has been described. The developed phantoms at 10% PVA-C and 1% cellulose are operative for microwave frequencies from 500 MHz to 20 GHz, as well as for US imaging, mimicking soft tissues in the selected range. The results provided by the microwave model properly fit the relative permittivities of soft tissues, enabling one to customize phantoms in the analyzed frequency band. Furthermore, the ultrasound properties of soft tissues can be simultaneously mimicked providing significant results in terms of acoustic velocity and attenuation.

The presented phantoms demonstrated feasibility of the employed materials as well as the fabrication process itself to develop customized, complex structures for clinical applications. These phantoms are unique tools for combined microwave and ultrasound applications, although the attenuation coefficient requires further adjustment to properly match soft tissues. Therefore, a broad range of applications are foreseen in this clinical domain.

## Data Availability

The authors declare that all relevant data are available within the paper.
